# Comparative Proteomics Analysis of Engineered *Saccharomyces cerevisiae* with Enhanced Biofuel Precursor Production

**DOI:** 10.1371/journal.pone.0084661

**Published:** 2013-12-23

**Authors:** Xiaoling Tang, Huixing Feng, Jianhua Zhang, Wei Ning Chen

**Affiliations:** School of Chemical and Biomedical Engineering, College of Engineering, Nanyang Technological University, Singapore, Singapore; Auburn University, United States of America

## Abstract

The yeast *Saccharomyces cerevisiae* was metabolically modified for enhanced biofuel precursor production by knocking out genes encoding mitochondrial isocitrate dehydrogenase and over-expression of a heterologous ATP-citrate lyase. A comparative iTRAQ-coupled 2D LC-MS/MS analysis was performed to obtain a global overview of ubiquitous protein expression changes in *S. cerevisiae* engineered strains. More than 300 proteins were identified. Among these proteins, 37 were found differentially expressed in engineered strains and they were classified into specific categories based on their enzyme functions. Most of the proteins involved in glycolytic and pyruvate branch-point pathways were found to be up-regulated and the proteins involved in respiration and glyoxylate pathway were however found to be down-regulated in engineered strains. Moreover, the metabolic modification of *S. cerevisiae* cells resulted in a number of up-regulated proteins involved in stress response and differentially expressed proteins involved in amino acid metabolism and protein biosynthesis pathways. These LC-MS/MS based proteomics analysis results not only offered extensive information in identifying potential protein-protein interactions, signal pathways and ubiquitous cellular changes elicited by the engineered pathways, but also provided a meaningful biological information platform serving further modification of yeast cells for enhanced biofuel production.

## Introduction

The biofuel produced from microorganisms is playing a more and more important role in modern society. Its metabolic process involves a large number of protein functions and mysterious behaviors of cellular responses to environmental conditions [1–3]. The production level of biofuel precursors like fatty acids in microorganisms is different from species such as oleaginous and non-oleaginous microbes. It also changes under different culture conditions [4,5]. A better understanding of the biofuel production process in microorganisms is beneficial to provide a biological information platform serving modification of microbes for biofuel production improvement. Based on the well known genetic information and metabolic pathways, *Saccharomyces cerevisiae* serves as an excellent model in improving the understanding of biofuel metabolism. With the development of modern metabolic and genetic technologies, its biofuel metabolic pathways can be easily regulated. 

The citrate generated from the citric acid cycle (TCA cycle) is a prominent intermediate in both lipid and carbohydrate synthesis. In yeast, the citrate can be accumulated by regulating the TCA cycle and transported out to cytosol. Under the enzyme of ATP-citrate lyase, it can be cleaved to acetyl-CoA, which is the key starting material for fatty acid synthesis [6]. In our previous study, the yeast *S. cerevisiae* was selected as a metabolic engineering model for enhanced fatty acids synthesis. The genes *idh1* and *idh2*, which encoded two subunits of mitochondrial isocitrate dehydrogenase, were disrupted to increase the intracellular citrate level. A heterologous ATP-citrate lyase, which did not exist in original *S. cerevisiae* cells, was cloned and expressed in the gene disrupted strain. Utilizing this engineered pathway, *S. cerevisiae* accumulated much more fatty acids, especially in terms of the long chain mono-unsaturated fatty acids [7].

Apparently, the activities of mitochondrial isocitrate dehydrogenase and cytosol ATP-citrate lyase are correlated with the onset of fatty acids accumulation in yeast. However, the metabolic modification of *S. cerevisiae* cells could result in carbon source utilization changes and energy transfer changes. Besides, the accumulated biofuel precursors generated from the engineered pathway could have a considerable solvent-related effect on the growth of *S. cerevisiae*. The analysis of proteomic changes between *S. cerevisiae* wild type and engineered strains could help us better understand the protein functions involved in specific metabolic pathways and the adjustment mechanism of *S. cerevisiae* cells to counteract with growth stress. 

The 2D LC-MS/MS based proteomics approach provides qualitative and quantitative analysis of global proteins in microorganism cells [8]. It is a common and useful tool for the systematic research. The application of the proteomics based systematic research could provide extensive information in identifying potential protein-protein interactions, signal pathways and in turn the possible mechanism of changes in biofuel production levels [9,10].

The present study focuses on the comparative proteomics analysis between *S. cerevisiae* wild type strain and engineered strains. The iTRAQ-coupled 2D LC-MS/MS analysis approach is applied to identify and quantify the proteins. This study not only offers a global overview of ubiquitous protein expression changes in *S. cerevisiae* modified cells but also provides meaningful information about the regulation mechanism of *S. cerevisiae* metabolic pathways.

## Materials and Methods

### Strains and Culture Conditions

The yeast expression vector pVTU260 was transformed into *S. cerevisiae* BY4741 to construct wt-pvtu strain, which was taken as the wild type. The *idh1* and *idh2* genes, which encoded two subunits of mitochondrial isocitrate dehydrogenase, were knocked out in *S. cerevisiae* BY4741 to construct ∆*idh1/2* strain and the pVTU260 vector was transformed into ∆*idh1/2* strain to construct ∆*idh1/2*-pvtu strain. A heterologous ATP-citrate lyase gene (*acl*) from *Mus musculus* (ACCESSION BC056378.1) was inserted into pVTU260 vector and transformed into ∆*idh1/2* to construct ∆*idh1/2*-*acl* strain. The detailed construction of the methods was made according to previous study [10]. 

Single colonies of wt-pvtu, ∆*idh1/2*-pvtu and ∆*idh1/2*-acl strains from agar plates were selected and cultured in 5 ml minimal glucose (YNB-URA) medium: 0.67 % yeast nitrogen base without amino acids and 2 % dextrose (wt/vol) supplemented with URA dropout amino acid mixture. When the cells reached to growth phase, each of 10 OD cells were injected into 50 ml minimal glucose (YNB-URA) medium respectively. All of the cultures were kept at 30 °C and at a centrifugation speed of 250 rpm.

### Protein Extraction, Digestion and Labeling

Same amount (20OD) of the yeast cells were collected and centrifuged at 12000 rpm for 5 min to remove the supernatant medium. The cells were then washed twice with distilled water and re-suspended in 500 μl yeast lysis buffer, which contained 50 mM HEPES, 1 mM DTT, 1 mM PMSF, 1 mM EDTA and 5 % glycerol. An equal volume of glass beads (425-600 μm) was added into the sample tubes. The mix was performed in the bead mill: the vortex speed was set at 4.0 m/s and the vortex time was set as 20 s for one cycle, total of 5 cycles were carried out. After vortex, samples were centrifuged at 12000 rpm for 30 min at 4 °C and the supernatants were then collected. The protein quantification was determined following the protocol of 2-D Quant Kit (GE Healthcare). A standard curve of BSA concentration was plotted using the same method.

A total of 100 µg proteins from wt-pvtu, ∆*idh1/2*-pvtu and ∆*idh1/2*-acl cells were collected to generate iTRAQ labeled peptides. The proteins were precipitated by addition of 4 volumes of cold acetone at −20 °C for 2 hours. The precipitated samples were carefully centrifuged at 12000 rpm for 5 min and the acetone was removed. To each sample tube, 20 µl dissolution buffer and 1µl denaturant contained in iTRAQ Reagent Multiplex Kit (Applied Biosystems, CA) were added and mixed, followed by adding 2 µl reducing reagent. The tubes were then incubated at 60 °C for 1 h. After incubation, 1 µl cysteine blocking reagent was added and the tubes were kept at room temperature for 10 min. After digestion with 20 µl of 0.25 µg/µl sequence grade modified trypsin (Promega, US) at 37 °C overnight, samples from wt-pvtu, ∆*idh1/2*-pvtu and ∆*idh1/2*-*acl* were labeled with iTRAQ tag 114, 116 and 117, respectively. The labeled samples were then mixed together and condensed to 1 μg/μl at final concentration roughly. 

### On-line 2D Nano LC-MS/MS Analysis

The labeled samples were performed on the online 2D Nano-LC-MS/MS system. An Agilent 1200 Series LC system, interfaced with a 6530 Q-TOF mass spectrometer was applied for the analysis.

In the first dimension, 4 μg of combined iTRAQ labeled peptides mixture was loaded onto the system and separated by a strong cation exchange (SCX) nano-column (0.32 × 50 mm, 5 µm) (Agilent Technologies, CA). The peptides were eluted stepwise by injecting gradient ammonium formate solution with different concentrations of 20, 40, 60, 80, 100, 200, 500 and 1000 mM. The diagrams of total intensity chromatogram results of peptides eluted by gradient concentrations of ammonium formate and the representative MS spectrum showing selected peptide precursors were shown in Figure S1 and Figure S2 in File S1.

In the second dimension, the peptides that sequentially eluted from the SCX column were trapped in a Zorbax C18 enrichment column (0.3 ×5 mm, 5µm) and washed isocratically with 97% solvent A (water with 0.1% formic acid) and 3% solvent B (acetonitrile with 0.1% formic acid) at a flow rate of 4 μl/min. The eluted peptides were then bound on a C18 reverse phase column integrated with a nano-LC-Chip and eluted with 95% solvent A (water with 0.1 % formic acid) and 5% solvent B (acetonitrile with 0.1% formic acid) at a flow rate of 3 μl/min over 45 min. After that, the flow was kept at 90% B for 5 min and then brought to initial state in 10 min for reconditioning the column. Altogether 9 runs were performed to finish one round of detection. 

A 6530 Q-TOF mass spectrometer, interfaced with a Nano-LC-Chip Cube nanospray source was applied for electrospray analysis. The Agilent Masshunter Workstation Q-TOF was applied for obtaining MS data. Survey scans were acquired from *m/z* 450-1500 at 3 Hz, with up to two precursors selected for MS/MS from *m/z* 50-2000 at 2 Hz. 

### LC-MS/MS Data Analysis

The peptide identifications were performed using Spectrum Mill system (Agilent Technologist, CA). It was compared and analyzed through the UniprotKB/Swiss-Prot database (Geneva, Switzerland). Each MS/MS spectrum was searched for the species of *S. cerevisiae*. The iTRAQ reagent labels were allowed by the database and the modification was fixed under the situation of methylmethanethiosulfate-labeled cysteine, plus two missed cleavages. The mass tolerance for peptide identification was ± 2.5 Da for MS and ± 0.7 Da for MS/MS. The high confidence for searched proteins was carried out by auto-validation. The quantification of proteins in the case of iTRAQ was showed in peak area ratios of 116/114 and 117/114. The overlapping isotopic contributions were considered as the correction of calculated peak area ratios, which was used to the estimation of relative abundances of a specific peptide. The standard deviation and confidence value for each average peak area ratio were calculated and the protein quantification data with confidence > 95 %, p value < 0.05 were selected for further analysis.

### Real-Time RT-PCR Investigation

The RNA of wt-pvtu, ∆*idh1/2*-pvtu and ∆*idh1/2*-acl cells was extracted by RNeasy Mini kit (QIAGEN, USA) under the method of RNA extraction of yeast. The quantification of RNA was calculated through NanoDrop 2000C system (Thermo scientific, USA) and different samples were diluted to the same concentration as templates. 

The primers of selected proteins from *S. cerevisiae* strain were synthesized by 1st Base Pte Ltd (Singapore). According to the protocol provide by iScript one-step RT-PCR kit (Bio-Rad, USA), the Real-time RT-PCR mix was prepared and analyzed by an IQ5 multicolor Real-time RT-PCR detection system (Bio-Rad, USA). The Real-time RT-PCR cycling program was: 50 °C for 10 min, 95 °C for 5 min and then 45 cycles as the following: 95 °C 10 s, 55 °C 30 s. The melt curve analysis was carried out within 80 cycles as the following: 95 °C 60 s, 55 °C 60 s, 55 °C 10s. The disassociation analysis was routinely carried out by acquiring fluorescent reading for 0.5 °C increase from 55 °C to 95°C 

### GC-MS Preparation and Analysis

Same amounts (20 OD) of wt-pvtu, ∆*idh1/2*-pvtu and ∆*idh1/2*-acl cells were collected and centrifuged at 12000 rpm for 5 min to remove the supernatant. The cell pellets were then washed twice with distilled water and re-dissolved in 800 μl pure methanol. 10 μl ribitol (2 mg/ml) was added as internal standard. Each sample tube was performed on an ultrasonic processor. The amplitude was set at 50% and the sonic time was set at 0.3 s per cycle. Total of 60 cycles were carried out. After sonication, the sample tubes were centrifuged at 12000 rpm for 10 min and the clear supernatants were collected and evaporated to complete dryness at room temperature. 

The derivatization was carried out by adding 50 μl 20 mg/ml of methoxyamine hydrochloride in pyridine at 37 °C for 1 h. 99 μl MSTFA and 1 μl TMCS were added to above sample tubes for silylation at 70 °C for 30 min. After incubation, the prepared samples were then shaken at room temperature for 1h and then ready for the GC-MS analysis. The GC-MS analysis was carried out according to previous procedure and the results were analyzed by both comparing with standard and searching from the metabolite spectra database.

## Results

The previous study has indicated that the ∆*idh1/2* strain accumulated much more citrate than the wild type. Over-expression of a heterologous ATP-citrate lyase in this double disrupted strain resulted in enhancement of fatty acid production, especially in terms of long chain mono-unsaturated fatty acids. However, the biomass of ∆*idh1/2*-*acl* strain decreased and both the ATP and NADPH levels were lower than that in wt-pvtu strain. Herein, the proteomics analysis was carried out among wt-pvtu, ∆*idh1/2*-pvtu and ∆*idh1/2*-*acl* strains. The protein expression differences among these strains were investigated. The intracellular proteomics profile can reveal the metabolic flux mechanism in *S. cerevisiae* strains with modified metabolic pathways.

### Differentially Expressed Proteins among wt-pvtu, ∆*idh1/2*-pvtu and ∆*idh1/2*-*acl* Strains

The On-line 2D LC-MS/MS system was applied to analyze the proteome differences among wt-pvtu, ∆*idh1/2*-pvtu and ∆*idh1/2*-*acl* strains. The Spectrum Mill system was used for the peptide identifications. One selected peptide fragmentation spectrum of the protein triosephosphate isomerase was shown in Figure S3 in File S1. Based on the analysis method, a total of more than 300 proteins were detected. The protein identification levels were based on ProtScore with an Unused protein score more than 2.0 and with a confidence value of > 95 %, whereas, the proteins with ProtScore less than 2.0 were not selected for the analysis. 

Among these detected proteins, 37 differentially expressed ones were found and classified into specific categories based on their enzyme functions (Table 1). It was noteworthy that five proteins (PGI1, TPI1, TDH2, GPM1, PYK1) that played a role in glycolytic pathway, and three proteins (PDC1, ADH1 and PYC2) that were responsible for pyruvate branch-point pathways were up-regulated in both ∆*idh1/2*-pvtu and ∆*idh1/2*-*acl* strains. The proteins PDA1 and ACO1 involved in the TCA cycle showed up-regulation in both ∆*idh1/2*-pvtu and ∆*idh1/2*-*acl* strains, whereas the protein SDH1, which was not detected in ∆*idh1/2*-pvtu, showed down-regulation in ∆*idh1/2*-*acl* strain. The proteins ICL1 and MLS1 involved in glyoxylate cycle showed down-regulation in ∆*idh1/2*-pvtu and ∆*idh1/2*-*acl* strains.

**Table 1:** Relative changes in protein expression between *S. cerevisiae* wild type and engineered strains.

**Protein**	**Description**	**No. of peptides**	**Average of A/C**	**Average of B/C**
**Glycolysis**				
PGI1	Glucose 6-phosphate isomerase	19	1.44±0.014	1.46±0.205
TPI1	Triosephosphate isomerase	15	1.80±0.021	2.50±0.460
TDH3	Glyceraldehydes-3-phosphate dehydrogenase 3	24	1.43±0.036	1.60±0.071
GPM1	Phosphoglycerate mutase	19	1.45±0.010	1.48±0.216
PYK1	Pyruvate kinase	35	1.34±0.022	1.45±0.004
**Pyruvate branch-point**				
PDC1	Pyruvate decarboxylase isozyme 1	26	1.35±0.002	1.65±0.057
ADH1	Alcohol dehydrogenase 1	15	1.57±0.345	1.60±0.004
PYC2	Pyruvate carboxylase 2	2	1.21±0.107	1.30±0.176
**TCA cycle**				
PDA1	Pyruvate dehydrogenase E1 component subunit	3	1.39±0.300	1.54±0.106
ACO1	Aconitate hydratase	19	1.75±0.132	2.48±0.113
SDH1	Succinate dehydrogenase	2	--	0.55±0.190
**Glyoxylate cycle**				
ICL1	Isocitrate lyase	6	0.68±0.019	0.77±0.028
MLS1	Malate synthase	6	0.62±0.105	0.53±0.007
**Glycogenesis**				
UGP1	UTP—glucose-1-phosphate uridylyltransferase	2	1.77±0.058	1.84±0.064
**Respiration**				
MCR1	NADH-cytochrome b5 reductase 2	4	0.55±0.026	0.58±0.035
GPD1	Glycerol-3-phosphate dehydrogenase 1	3	0.43±0.019	0.27±0.011
AAC2	ADP, ATP carrier protein 2	2	0.79±0.113	0.73±0.060
**Antioxidants**				
TRX2	Thioredoxin-2	6	1.57±0.024	1.83±0.148
GRX2	Glutaredoxin 2, mitochondrial	5	--	1.46±0.032
AHP1	Peroxiredoxin type 2	12	1.04±0.018	1.76±0.265
**Amino-acid metabolism**				
ASN1	Asparagine synthetase	5	1.99±0.488	2.86±0.672
HOM2	Asparatate-semialdehyde dehydrogenase	3	1.28±0.045	1.33±0.138
AAT2	Aspartate aminotransferase	2	--	1.55±0.074
HOM6	Homoserine dehydrogenase	10	1.31±0.010	1.26±0.014
SHM2	Serine hydroxymethyltransferase	9	0.51±0.150	0.57±0.042
ARG1	Argininosuccinate synthase	3	--	1.84±0.251
**Protein biosynthesis**				
YEF3	Elongation factor 3A	6	1.21±0.004	1.71±0.042
RPL3	60s ribosomal protein L3	4	1.35±0.128	1.49±0.057
RPL8B	60s ribosomal proteion L8-B	3	1.6±0.007	1.84±0.360
RPS0A	40s ribosomal protein S0-A	3	1.12±0.031	1.52±0.219
RPS5	40s ribosomalprotein S5	2	1.42±0.111	1.55±0.060
**Heat shock proteins**				
SSA1	Heat shock protein SSA1	32	1.02±0.001	1.55±0.085
HSP26	Heat shock protein 26	12	0.99±0.005	1.60±0.039
**Others**				
MPG1	Mannose-1-phosphate guanyltransferase	3	1.43±0.104	1.54±0.173
GPP1	(DL)-glycerol-3-phosphatase 1	6	0.99±0.011	1.36±0.095
ADE13	Adenylosuccinate lyase	3	1.31±0.034	1.76±0.085
INO1	Inositol-3-phosphate synthase	5	0.77±0.015	0.75±0.028

The “Average of A/C” refers to the average ratio of protein expression level in ∆*idh1/2*-pvtu strain over that in wt-pvtu strain and “Average of B/C”refers to the average ratio of protein expression level in ∆*idh1/2*-*acl* strain over that in wt-pvtu strain. Herein, each protein expression level in wt-pvtu was taken 100% and the deviation was calculated from three independently LC-MS/MS analysis results. Several proteins that were not detected in ∆*idh1/2*-pvtu strain was indicated by “--”.

Five proteins involved in stress response were also found differentially expressed in ∆*idh1/2*-*acl* strain. These proteins included two heat shock proteins (SSA1, HSP26), one peroxiredoxin (AHP1), one glutaredoxin (GRX2) and one thioredoxin (TRX2). Compared with that in wt-pvtu strain, most of the stress response related proteins showed more than 1.5- fold increase in ∆*idh1/2*-*acl* strain. However, the heat shock proteins SSA1, HSP26 and peroxiredoxin AHP1 in ∆*idh1/2*-pvtu strain showed no expression difference.

Three proteins (MCR1, GPD1, and AAC2) involved in respiration pathway were identified as down-regulation proteins in ∆*idh1/2*-pvtu and ∆*idh1/2*-*acl* strains. Moreover, various proteins involved in protein biosynthesis and amino acid metabolism were also found differentially expressed between wild type and engineered strains. 

### Real-Time RT-PCR Based mRNA Levels Investigation of Differentially Expressed Proteins

**Figure 1:**
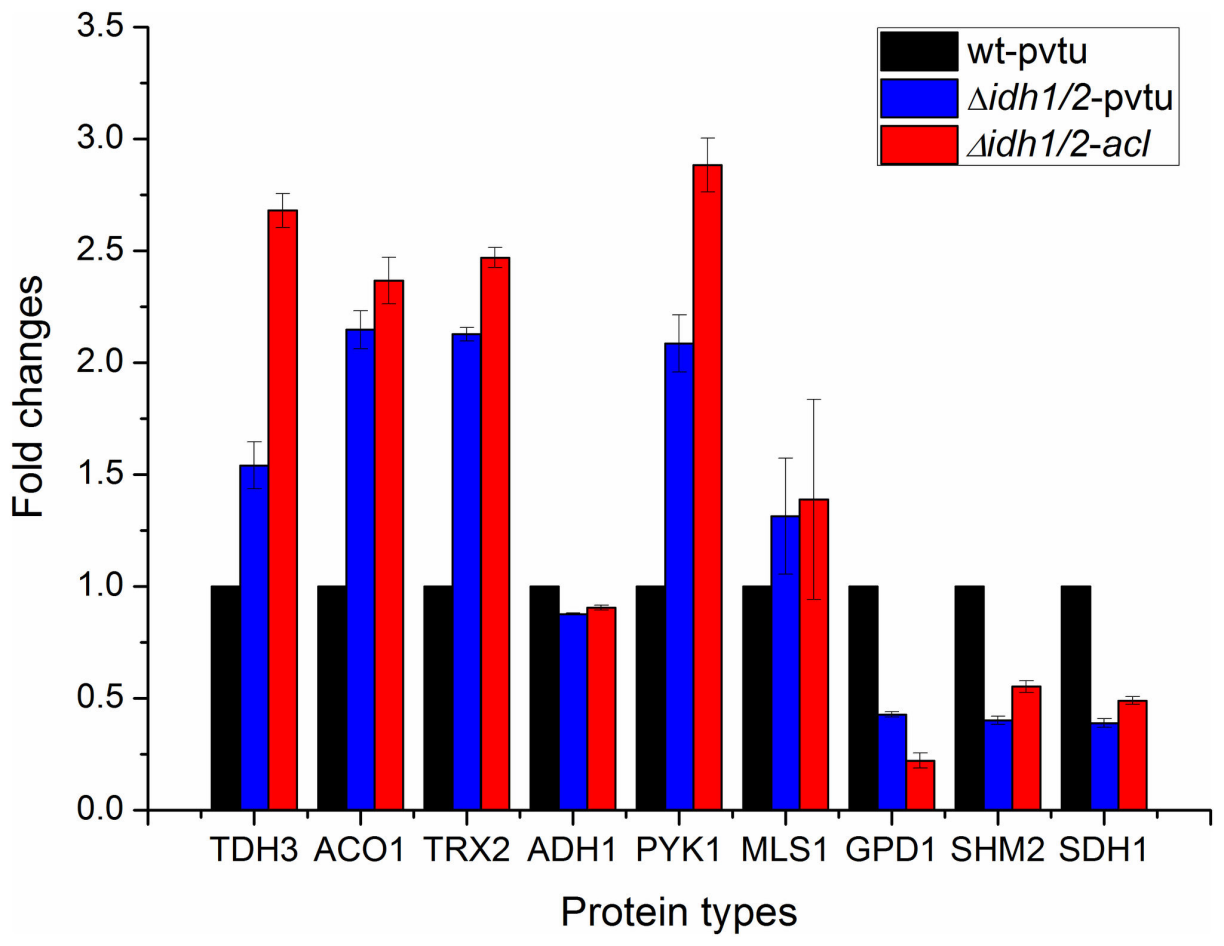
Real-time RT-PCR analysis of mRNA levels of selected differentially expressed proteins among wt-pvtu, ∆*idh1/2*-pvtu and ∆*idh1/2*-*acl* strains. Three independent experiments were carried out. The mRNA levels in wt-pvtu strain were taken as controls (100%) and mRNA levels in ∆*idh1/2*-pvtu and ∆*idh1/2*-*acl* strains were being ratio to that in the wt-pvtu strain.

The mRNA played an important role as protein precursor and its cellular level could be a reflector of protein expression result. In order to examine the mRNA levels of differentially expressed proteins from LC-MS/MS results among wt-pvtu, ∆*idh1/2*-pvtu and ∆*idh1/2*-*acl* strains, the Real-time RT-PCR procedure was performed on 9 selected proteins (Table 2). The S. cerevisiae actin (ACT1) was selected as the endogenous control. The final products length was controlled between 100-250 bp. In each strain, the total mRNA was extracted from three independent culture batches of cells and used as templates at the same concentration. The mRNA levels of proteins in wt-pvtu strain were taken as controls (100%), and the mRNA levels of the same proteins in ∆*idh1/2*-pvtu and ∆*idh1/2*-*acl* strains were being ratio to that in the wt-pvtu strain. The results were shown in Figure 1. The representative Real-time RP-PCR reaction curves generated by the IQ5 multicolor Real-time RT-PCR detection system was shown in Figure S4 in File S1. The results from Figure 1 showed that for most of the proteins, the changing trends exhibited in the mRNA levels were consistent with the changing trends in protein expression levels. In both of the ∆*idh1/2*-pvtu and ∆*idh1/2*-*acl* strains, the mRNA levels of TDH3, ACO1, TRX2 and PYK1 showed similar up-regulation and the mRNA levels of SHM2, GPD1, SDH1 showed similar down-regulation as protein levels from the LC-MS/MS result. However, the changing trends which showed inconsistency between Real-time RT-PCR and LC-MS/MS result were found in proteins MLS1 and ADH1. For MLS1, it showed up-regulation in the mRNA level, but was demonstrated as down-regulation protein, whereas for ADH1, it showed down-regulation in the mRNA level, but was demonstrated as up-regulation protein in ∆*idh1/2*-pvtu and ∆*idh1/2*-*acl* strains. Such kind of variances could be explained by the phenomenon of post-translational modification in eukaryotic cells, such as acetylation, amidation, methylation and phosphorylation.

**Table 2:** Primer sequences for real-time RT-PCR.

**Protein name**	**Sense primer**	**Antisense primer**	**PCR product (bp)**
TDH3	5’ACGATGACAAGCACATCATC3’	5’ATGTGCTTTTGAGCAGTGTC3’	151
ACO1	5’AGCAACTGGTCGTGGTAAGA3’	5’TGACATCCAATGGCCAGTTA3’	216
TRX2	5’ATACGACAGTGCTTTAGCAT3’	5’TAGGCATGGAAGAAACTTCA3’	194
ADH1	5’CACGAAGGTGCCGGTGTCGT3’	5’AGAACCGTCGTGGGTGTAAC3’	177
PYK1	5’TGGACGACGGGATTCTCTCT3’	5’GACTCCGAATTGCAAGTCCT3’	176
MLS1	5’CCAAAGATGGAGCACCACTT3’	5’AGTCCCAACGTCCGCAATTC3’	196
SHM2	5’TGTTTACTCCGCTATCATGA3’	5’TTGGCCAATACTTGCAAGTT3’	198
GPD1	5’AACATTGCCACCGAAGTCGC3’	5’CAACAACGTTCTTCAAAGCA3’	199
SDH1	5’CTTTGCCCTCGATCTGTTGA3’	5’ATGCTCTACCATAGCCACCA3’	131
ACT1	5’ AACTTTCAACGTTCCAGCCT3’	5’ CCACGTTCACTCAAGATCTT3’	216

### Intracellular Metabolites Production Levels among wt-pvtu, ∆*idh1/2*-pvtu and ∆*idh1/2*-*acl* Strains

**Figure 2:**
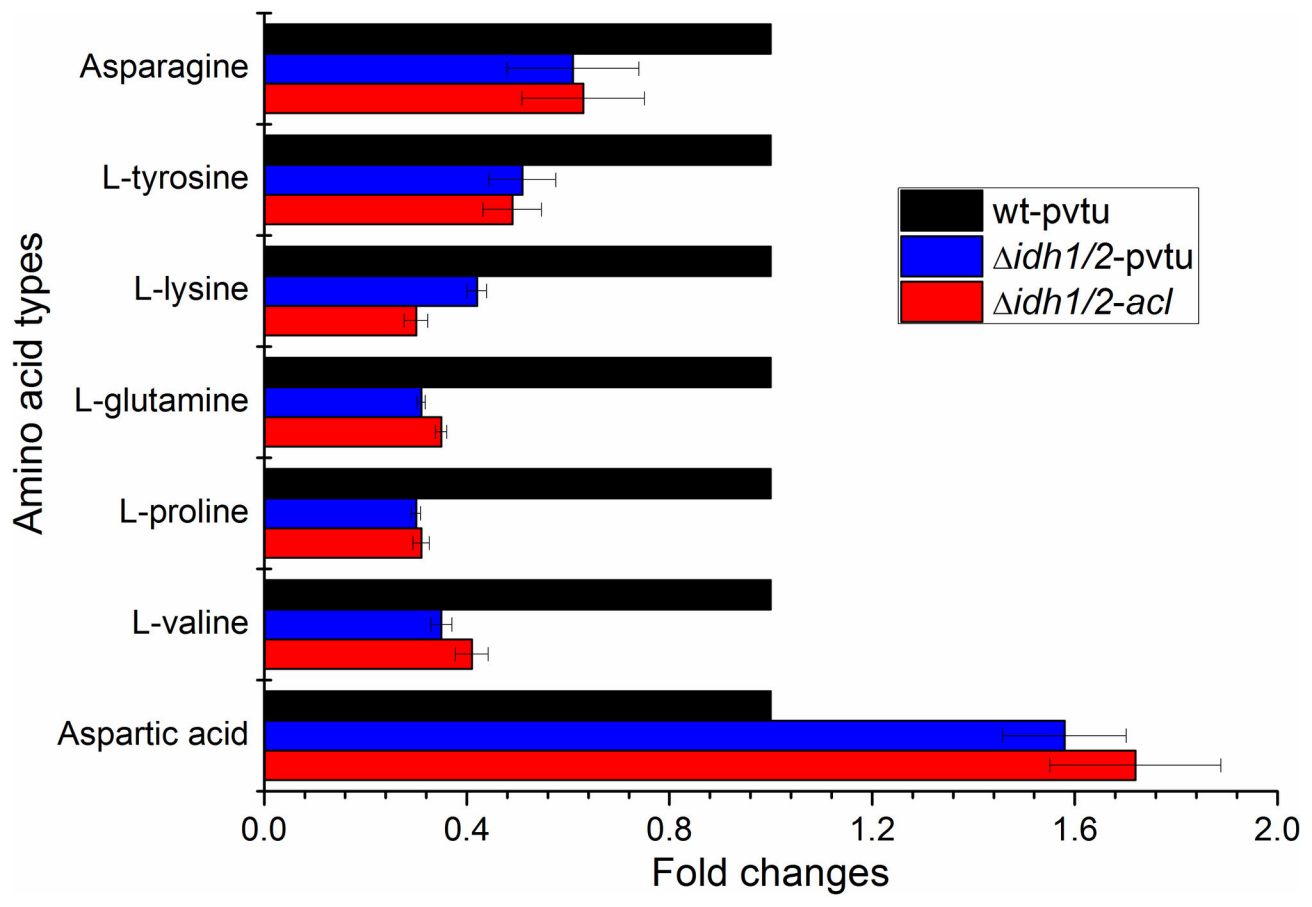
Differential production levels of intracellular amino acids among wt-pvtu, ∆*idh1/2*-pvtu and ∆*idh1/2*-*acl* strains. Three independent experiments were carried out. The amino acid levels in wt-pvtu strain were taken as controls and the amino acid levels in ∆*idh1/2*-pvtu and ∆*idh1/2*-*acl* strains were calculated accordingly.

**Figure 3:**
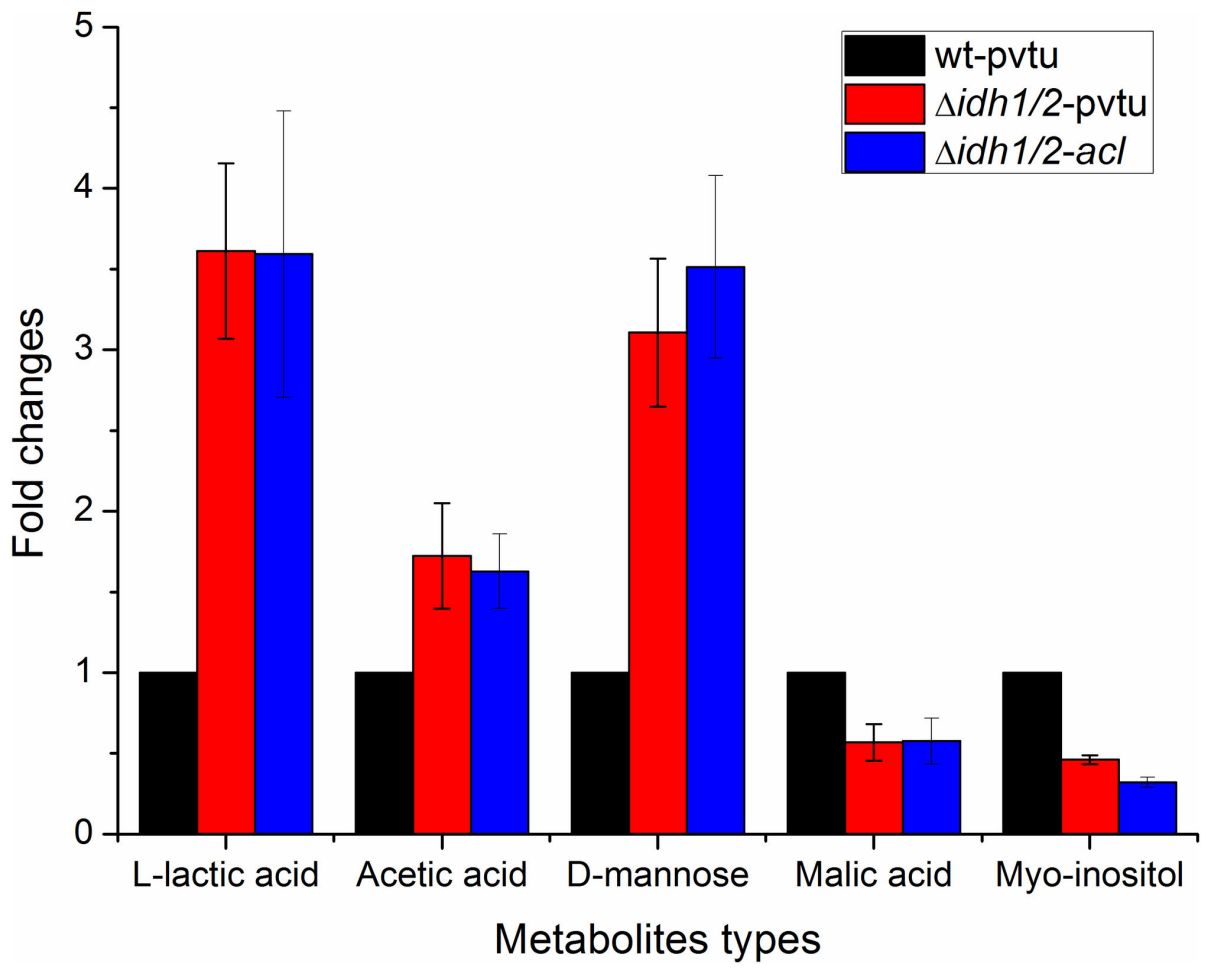
Differential production levels of intracellular metabolites among wt-pvtu, ∆*idh1/2*-pvtu and ∆*idh1/2*-*acl* strains. Three independent experiments were carried out. The metabolites production levels in wt-pvtu strain were taken as controls and the metabolites production levels in ∆*idh1/2*-pvtu and ∆*idh1/2*-*acl* strains were calculated accordingly.

The GC-MS analysis system was performed for intracellular metabolites identification and quantification among wt-pvtu, ∆*idh1/2*-pvtu and ∆*idh1/2*-*acl* strains. A number of metabolites were identified from the NIST library and SHIMADZU GC/MS Metabolite Mass Spectra Database. Herein, the production levels of metabolites in wt-pvtu strain were taken as controls, and the production levels of the same components in ∆*idh1/2*-pvtu and ∆*idh1/2*-*acl* strains were calculated accordingly. The results were shown in Figure 2 and Figure 3.

Among the detected metabolites, various amino acids were found differentially produced between *S. cerevisiae* wild type and engineered strains. These amino acids included L-valine, L-proline, L-lysine, L-glutamine, Asparagine, Aspartic acid and L-tyrosine (Figure 2). Except for the amino acid Aspartic acid, others showed down-regulation in ∆*idh1/2*-pvtu and ∆*idh1/2*-*acl* strains. Moreover, the metabolites lactic acid, acetic acid, d-mannose showed up-regulation in ∆*idh1/2*-pvtu and ∆*idh1/2*-*acl* strain, whereas, the metabolites myo-inositol and malic acid showed down-regulation in both of these two engineered strains (Figure 3). 

## Discussion

**Figure 4:**
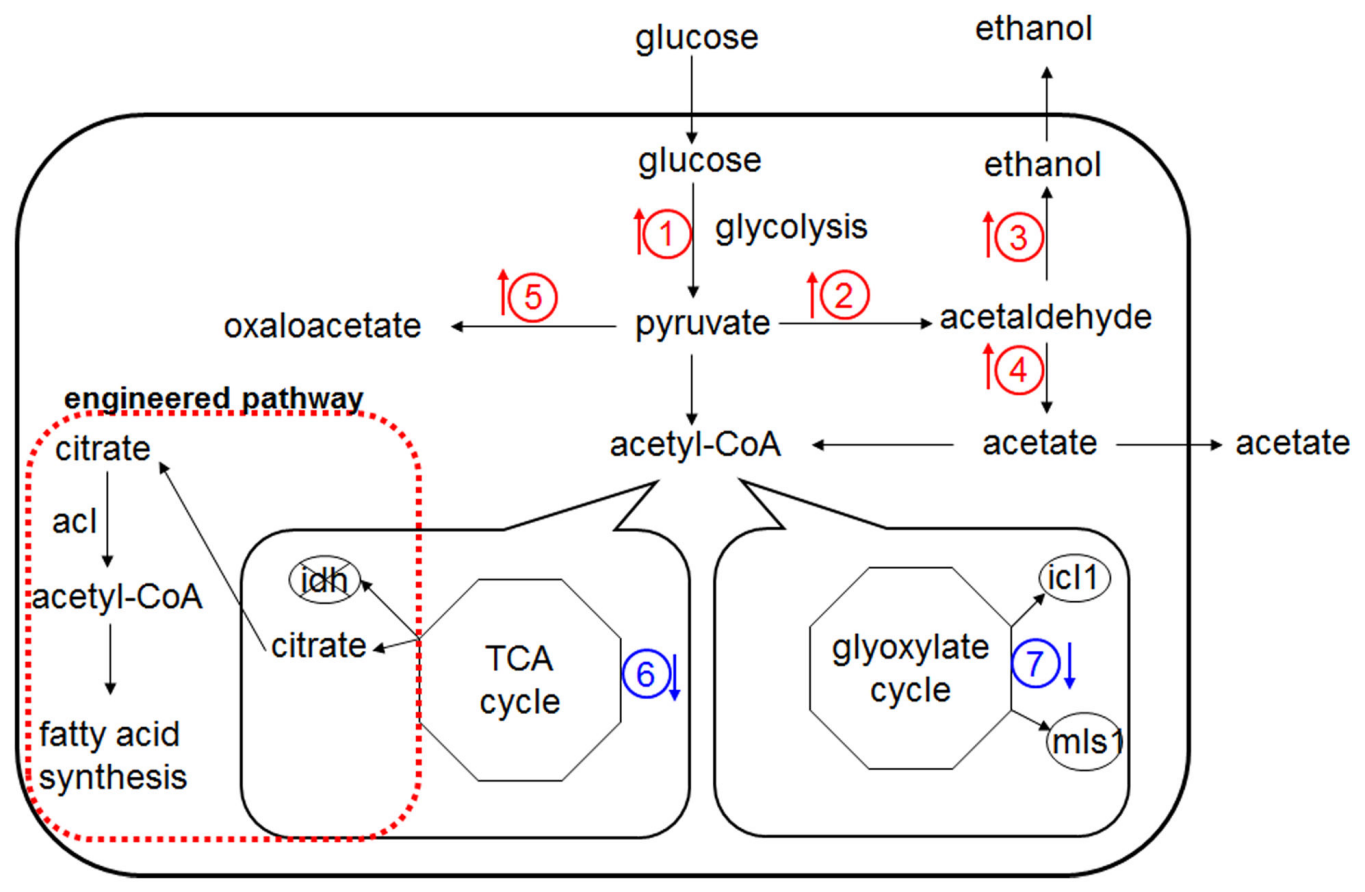
Schematic diagram of metabolic pathway changes in *S*. ***cerevisiae* engineered strains**. The engineered pathway in yeast with enhanced fatty acid synthesis was indicated by red dashed box. Both of the up-regulated and down regulated metabolic pathways in ∆*idh1/2*-*acl* strain were indicated by the different colored arrows. The proteins involved in other pathways that haven’t been detected from LC-MS/MS analysis were not shown in this diagram.

The LC-MS/MS approach enabled the analysis of relative protein expression levels among wt-pvtu, ∆*idh1/2*-pvtu and ∆*idh1/2*-*acl* strains. The metabolic engineering of *S. cerevisiae* resulted in significant changes in protein levels that were involved in crucial biological pathways (Figure 4).

During fermentative growth of *S. cerevisiae*, the glycolytic pathway plays an important role in energy and intermediates provision [11]. The results from Table 1 showed that the expression levels of five proteins (PGI1, TPI1, TDH3, GPM1 and PYK1) involved in glycolytic pathway were significantly increased in ∆*idh1/2*-pvtu and ∆*idh1/2*-*acl* strains. These proteins were responsible for converting glucose to pyruvate. Moreover, the PDC1 and ADH1 were two crucial enzymes involved in pyruvate-ethanol metabolic pathway and the PYC2 was involved in pyruvate-oxaloacetate metabolic pathway. Their expression levels also showed up-regulation in ∆*idh1/2*-pvtu and ∆*idh1/2*-*acl* strains. It could be explained that since the genes *idh1* and *idh2* were knocked out, the TCA cycle was definitely blocked. As a result, during its growth process, the ∆*idh1/2* strain would have preferred the fermentation process to the respiration process. Parts of the glucose were shifted from the TCA cycle to alcoholic fermentation and other by-pass pyruvate metabolic process. The study here was consistent with the previous report that during alcoholic fermentation process, enzymes involved in glycolytic pathway were much more efficiently expressed in yeast [12,13]. Besides, the higher amounts of acetic acid and lactic acid found in ∆*idh1/2*-pvtu and ∆*idh1/2*-*acl* strains indirectly verified this phenomenon (Figure 3). The glycolysis was a critical energy pathway and the enhanced glycolytic pathway in ∆*idh1/2* could provide energy compensation for the energy loss from the TCA cycle [14,15]. The comparison of protein expression levels between *S. cerevisiae* wild type and engineered strains provided useful information to regulate the sugar metabolism in the yeast cells.

Associated with the blockage of the TCA cycle, the proteins involved in the respiration pathway were found down-regulated in ∆*idh1/2*-pvtu and ∆*idh1/2*-*acl* strains. The expression levels of MCR1, which was involved in the electron transfer chain and GPD1, a major contributor of electrons to the electron transport chain in mitochondria showed to be much lower in ∆*idh1/2*-pvtu and ∆*idh1/2*-*acl* strains. The expression level of AAC2, which was taken as ADP, ATP carrier protein, also decreased in ∆*idh1/2* strains (Table 1). The result was consistent with previous result that ATP level was much lower in ∆*idh1/2* strains than that in wild type strain. These protein expression differences reflected the energy loss problem due to the blockage of the TCA cycle in ∆*idh1/2* strains. Since the fatty acid biosynthesis was an ATP-dependent process and restricted to the conditions of high energy load of the yeast cells, the biofuel precursor production was expected to be enhanced much more if the energy loss problem could be solved in the ∆*idh1/2*-*acl* strain. 

Interestingly, influenced by the modified pathways in *S. cerevisiae*, the proteins involved in glyoxylate cycle showed expression decrease in ∆*idh1/2*-pvtu and ∆*idh1/2*-*acl* strains. The ICL1 and MLS1 were two major enzymes involved in glyoxylate cycle and both of these two enzymes could be depressed by glucose [16]. There were research works which focused on coordinate regulation of the TCA cycle and glyoxylate cycle. The studies revealed that the enzymes in the TCA cycle had considerable effects on enzymes involved in glyoxylate pathways, regardless of their intracellular localization [17,18]. The enzymes ACO1, IDH1, and FUM1 involved in the TCA cycle were reported to be essential for acetate utilization and deleting one of these enzymes caused significant defect of the glyoxylate cycle in *S. cerevisiae*. This was again consistent with our results. Besides, the intracellular metabolites analysis results showed that the production level of malic acid was much lower in both of ∆*idh1/2*-pvtu and ∆*idh1/2*-*acl* strains. The malic acid could be produced from both the TCA cycle and glyoxylate pathway. Herein, the decreased amount of malic acid in ∆*idh1/2* cells was ideally correlated to the blockage of the TCA cycle and glyoxylate cycle. 

A number of proteins related to stress response were up-regulated in ∆*idh1/2*-*acl* strain (Table 1). These proteins included two heat shock proteins, one peroxiredoxin (AHP1), one glutaredoxin (GRX2) and one thioredoxin (TRX2). The accumulation of biofuels such as fatty acids-derived bioaldehyde, bioalkane or bioalcohols could generate solvent stress to yeast cells [19]. They could interfere with membrane protein function and result in an increased membrane permeability and membrane fluidity while diminishing the energy transduction [20,21]. The solvent stress from short-chain alcohols has been reported to have many parallels with heat shock in microorganisms [22] and one efficient way to solve the problem was to increase the expression levels of heat shock proteins. The up-regulation of heat shock proteins in ∆*idh1/2*-*acl* was a response to such a kind of solve stress. The proteomic results matched the previous explanation of why mono-unsaturated fatty acids accumulated in ∆*idh1/2*-*acl* strain: the shift of pyruvate metabolism from respiration to alcoholic fermentation resulted in increased production of ethanol and fatty acids, and this could generate solvent stress on *S. cerevisiae* cells. The mono-unsaturated fatty acids were essential components for membrane expansion in yeast cells [23]. The accumulation of mono-unsaturated fatty acids and up-regulation of heat shock proteins in ∆*idh1/2*-*acl* could be taken as an adjustment mechanism to counteract the membrane-fluidizing effects caused by solvent stress. 

The thioredoxin TRX2 was involved in many cellular processes, including oxidatively damaged proteins repair, protein folding, sulfur metabolism and redox homeostasis [24]. The peroxiredoxin AHP1 was identified as a key peroxidase involved in osmotic stress resistance and detoxification [25] and the glutaredoxin GRX2 was involved in reducing cytosolic protein- and non-protein-disulfides [26]. The up-regulation of these antioxidants in ∆*idh1/2*-*acl* could be beneficial for *S. cerevisiae* to counteract with the organic solvent stress, oxidative stress and repair oxidatively damaged proteins caused by the modified pathway. 

The amino acid metabolism was important for synthesis of unique proteins, peptides and other nitrogen-containing substances. They could be indirectly metabolized to release energy for the biological process [27]. The possible reason of difference in amino acids production level in ∆*idh1/2*-pvtu and ∆*idh1/2*-*acl* strains (Figure 2) was to keep them in balance in intracellular metabolism pathways. In addition, the protein GPP1 in yeast cells played an important role in osmo-adaptation [28]. The up-regulation of GPP1 in ∆*idh1/2*-*acl* but not in ∆*idh1/2*-pvtu could be due to the fact that the fatty acids accumulated in ∆*idh1/2*-*acl* strain but not in ∆*idh1/2*-pvtu strain. The MPG1 was involved in cell wall synthesis and it catalyzed alpha-D-mannose 1-phosphate to generate GDP-mannose [29]. The results from Figure 3 showed that the production level of D-mannose was higher in ∆*idh1/2*-pvtu and ∆*idh1/2*-*acl* strains, which was consistent with the increased protein expression level of MPG1. The protein INO1 was involved in myo-inositol biosynthesis process and the myo-inositol served as an important component in structural lipids synthesis in yeast [30]. The protein level of INO1 was down-regulated in ∆*idh1/2*-pvtu and ∆*idh1/2*-*acl* strain and this was consistent with the result of lower production level in cellular myo-inositol. The mechanism details of such expression and production changes in *S. cerevisiae* engineered strains however need to be further investigated.

## Conclusion

The comparative proteomics identification and quantification was performed among wt-pvtu, ∆*idh1/2*-pvtu and ∆*idh1/2*-*acl* strains. The proteins involved in various metabolic pathways were found to be differentially expressed and their functions were analyzed. The LC-MS/MS based proteomics analysis, together with GC-MS based metabolites analysis, not only offered an overview of ubiquitous cellular protein expression changes in *S. cerevisiae* engineered strains, but also provided a meaningful information platform which was beneficial for further modification of *S. cerevisiae* cells in terms of improving biofuel production.

## Supporting Information

File S1 **Figures S1-S4.**
**Figure S1.** Total intensity chromatogram results of peptides eluted by gradient concentrations of ammonium formate. **Figure S2.** (A) Representative MS spectrum showing selected four peptide precursors. (B) Representative MS/MS spectrum of one of the selected precursors. **Figure S3.** Representative peptide fragmentation spectrum of triosephosphate isomerase: FALGQGVGVILCIGETLEEK. **Figure S4.** Representative Real-time RT-PCR reaction figure of aconitate hydratase.
(DOC)
