# Supplementary material for: Comparative Proteomics Analysis of Engineered Saccharomyces cerevisiae with Enhanced Biofuel Precursor Production
Source: PLoS One. 2013 Dec 23;8(12):e84661. doi: 10.1371/journal.pone.0084661 (PMC3871657; doi:10.1371/journal.pone.0084661)
Supplement: File S1 — Figures S1-S4. Figure S1. Total intensity chromatogram results of peptides eluted by gradient concentrations of ammonium formate. Figure S2. (A) Representative MS spectrum showing selected four peptide precursors. (B) Representative MS/MS spectrum of one of the selected precursors. Figure S3. Representative peptide fragmentation spectrum of triosephosphate isomerase: FALGQGVGVILCIGETLEEK. Figure S4. Representative Real-time RT-PCR reaction figure of aconitate hydratase. (DOC) [file pone.0084661.s001.doc]

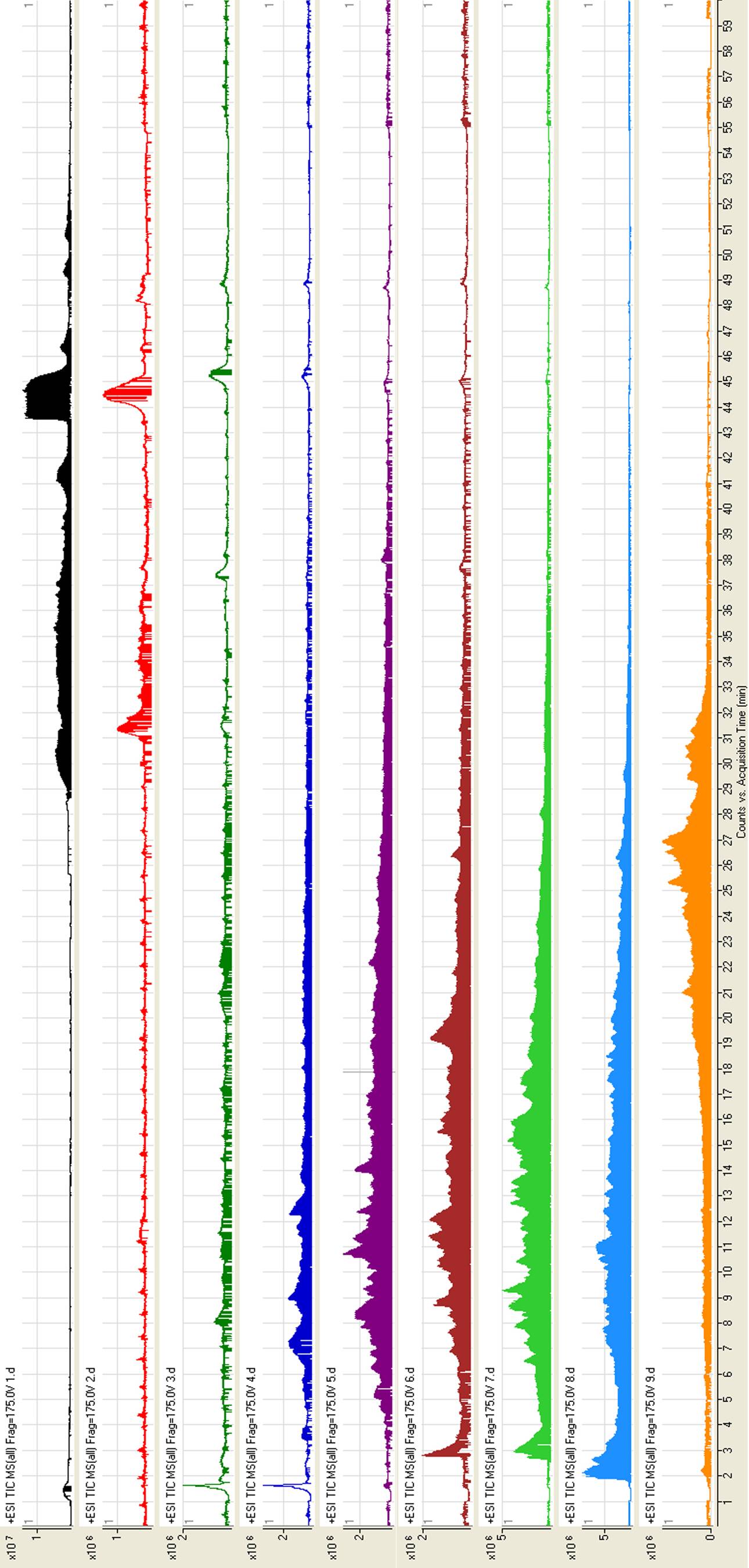


Figure S1. Total intensity chromatogram results of peptides eluted by gradient concentrations of ammonium formate.


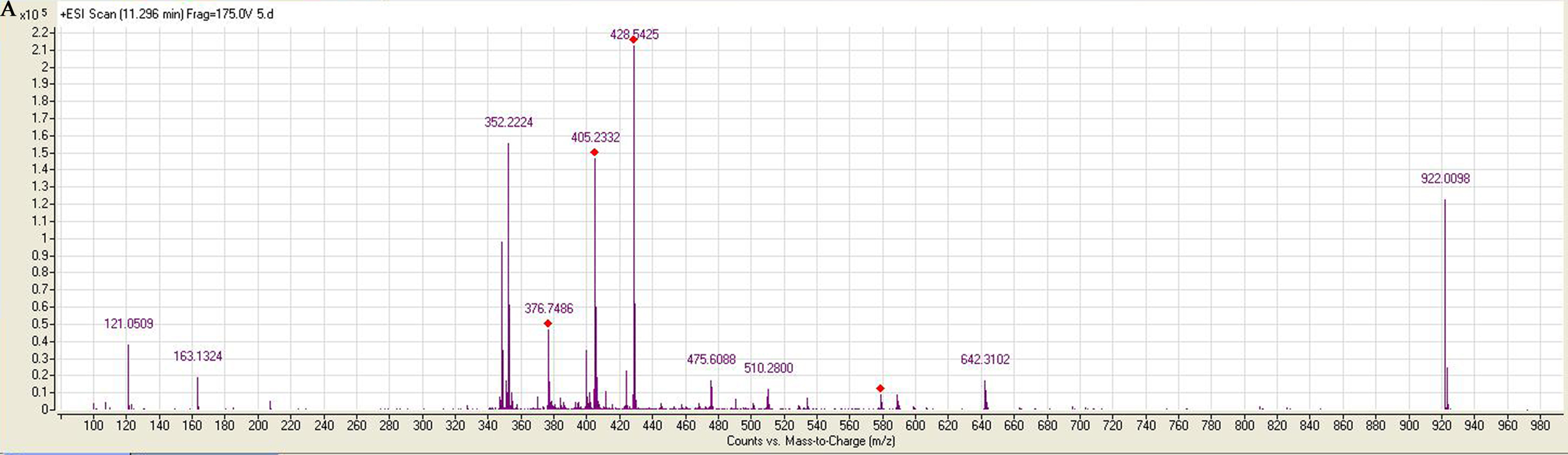

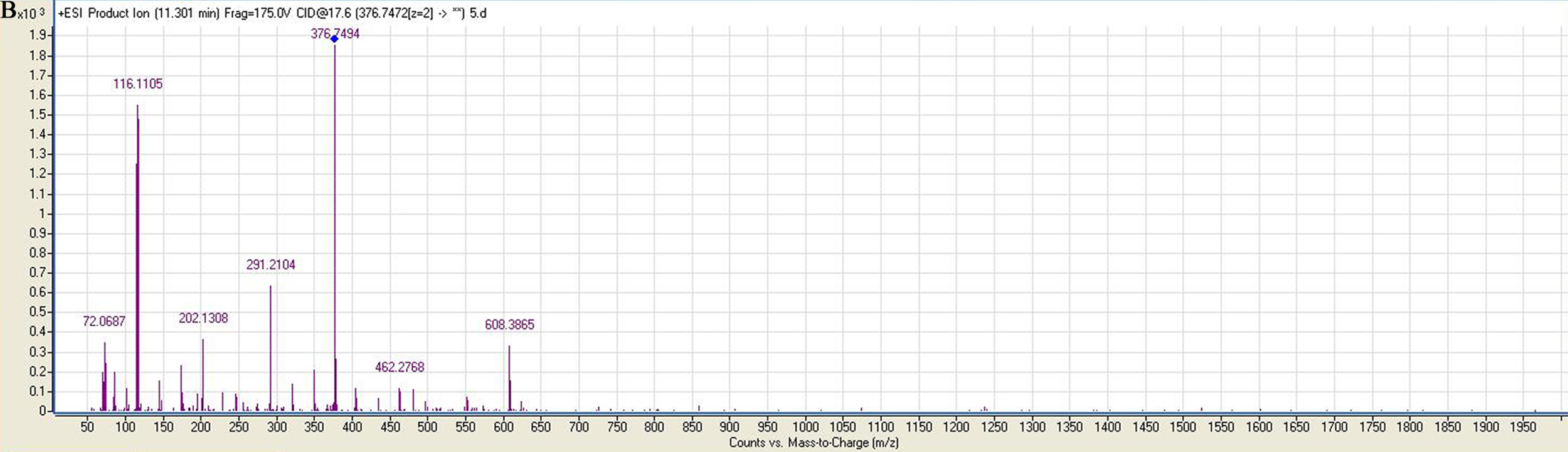


Figure S2. (A) Representative MS spectrum showing selected four peptide precursors (376.75, 405.23, 428.54 and 580) marked by red diamond. (B) Representative MS/MS spectrum of one of the selected precursors (376.75) marked by blue diamond.


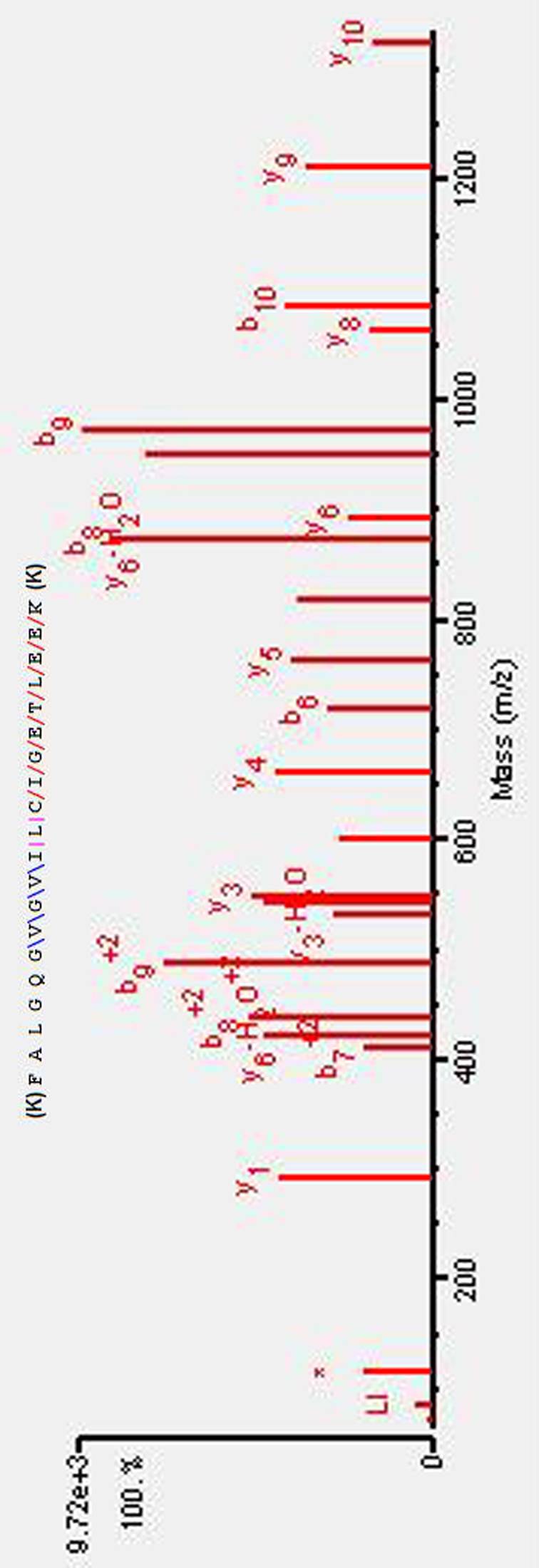


Figure S3. Representative peptide fragmentation spectrum of triosephosphate isomerase: FALGQGVGVILCIGETLEEK


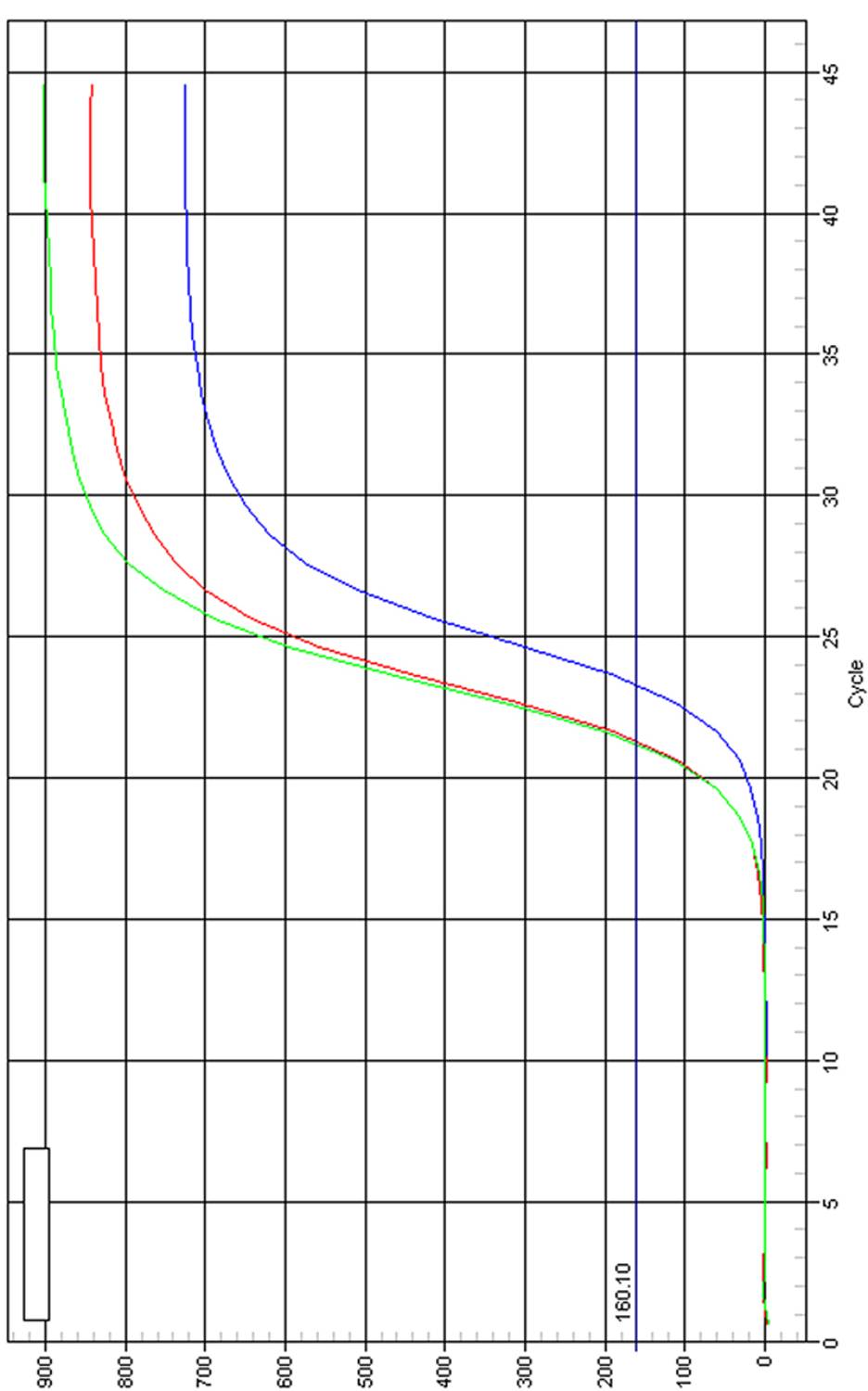


Figure S4. Representative Real-time RT-PCR reaction figure of aconitate hydratase. The X-axis represents the reaction cycle number and Y-axis represents the relative fluorescence units. The curve with blue color represents the result of wt-pvtu strain, the curve with red color represents the result of ∆*idh1/2*-pvtu strain and the curve with green color represents the result of ∆*idh1/2*-*acl* strain. The fold changes were calculated according to the following formula: ΔΔCt = SampleΔCt-ControlΔCt; the fold of sample vs control = 2^−ΔΔCt^, while the Ct represents the cycle number.
